# Effects of Expressive Writing on “Choking under Pressure” in High Test-Anxious Individuals

**DOI:** 10.3390/ijerph20010302

**Published:** 2022-12-25

**Authors:** Yuejin Yu, Xiaocong Zhang

**Affiliations:** 1 Department of Student Affairs Management, Nanjing Institute of Technology, Nanjing 211167, China; 2 Department of Psychology, School of Medicine and Holistic Integrated Medicine, Nanjing University of Chinese Medicine, Nanjing 210023, China

**Keywords:** expressive writing, test anxiety, working memory capacity, choking under pressure

## Abstract

(1) Background: High test-anxious students often fail to perform at their actual level and are prone to choking under pressure (CUP). The aim of the present study was to investigate whether expressive writing (EW) can help high test-anxious individuals reduce the degree of the CUP effect, and whether the intervention effects were different in people with different working memory capacities. (2) Methods: High test-anxious participants wrote expressively (EW group) or neutrally (control group) according to guidance, and then completed a modular arithmetic (MA) task under a high-stress condition. (3) Results: The state anxiety score of the control group was significantly higher than that of the EW group in the high-pressure situation, indicating that the EW intervention was helpful to alleviate the state anxiety. Subjects with high working memory capacity in the control group performed the complex MA task significantly less accurately in the high-stress situation than in the low-stress situation, showing the CUP effect. There was no significant difference in complex MA task scores between high- and low-stress situations for subjects with high working memory capacity in the EW group, indicating that the EW intervention can reduce the degree of the CUP effect. (4) Conclusions: EW intervention was effective in reducing state anxiety levels and attenuating the detrimental effects of test stress on cognitive processing in test-anxious individuals with high working memory capacity.

## 1. Introduction

Test anxiety is a prevalent psychological issue in a variety of educational settings [1,2]. Students have to experience a variety of evaluative situations such as written examinations, interviews, and public speaking. Examinations usually have significant impacts on students (e.g., their performance can affect the chances of receiving scholarships, further education, or employment) and constitute an important stressor for students [3]. Test anxiety can impair students’ academic performance and be detrimental to their physical and mental health [4,5]. Test-anxious students frequently underperform on some significant examinations and are more prone to choking under pressure (CUP) [6,7].

The CUP effect refers to the phenomenon whereby people underperform in high-stress situations relative to their performance without pressure [8,9]. According to the interference theory, the high-stress scenario induces a dual-task processing state, which is a significant contributor to the CUP effect [10]. High test-anxious individuals are more inclined to be concerned about items unrelated to the examination in high-stress test scenarios (i.e., task-irrelevant thinking). Specifically, task-irrelevant thinking comprises intrusive thinking, which is characterized by worrying about test results and others’ evaluations; and avoidant thinking, which is characterized by inhibiting and countering painful experiences [11]. In accordance with the interference theory, task-irrelevant thinking competes for cognitive resources with the current processing task and interferes with the working memory function of test-anxious individuals, which in turn impairs cognitive processing efficiency and performance [12]. With its constrained resources, the working memory system is responsible for maintaining information relevant to the current processing task and inhibiting task-irrelevant information [13]. The more working memory resources are consumed by the task-irrelevant thinking, the greater the interference of test stress on processing efficiency and performance [14].

People with different working memory capacities respond differently in high-pressure situations [15]. Individuals with high working memory capacity (HWMs) are more likely to experience the CUP effect than those with low working memory capacity (LWMs) [16]. HWMs typically utilize highly cognitively demanding strategies to solve problems, whereas LWMs tend to use low cognitively demanding but low-accuracy strategies [17]. Using highly cognitively demanding strategies makes HWMs outperform LWMs. However, the cognitive strategies utilized by HWMs are rendered useless if pressure-induced working memory consumption denies the capacity they typically depend on. When taking on tasks that require massive working memory resources in a high-pressure situation, HWMs perform even worse than LWMs [18].

Expressive writing (EW) is an emotional regulation technique that encourages individuals to write about their thoughts and feelings regarding an important stressor they are facing [19,20]. Written content typically contains a high proportion of emotion words (e.g., sad, depressed, distressed) and cognitive words (e.g., because, hence, think, consider) [21,22]. EW interventions have emotionally cathartic and cognitive adjustment functions, which could help test-anxious individuals reduce task-irrelevant thinking [23]. Studies have shown that writing down feelings and thoughts about forthcoming exams can aid students in reducing depression and anxiety [24], enhance working memory capacity [25,26], and improve test performance [27]. High test-anxious individuals can write to vent negative emotional experiences about exams, reduce stressful experiences, and relieve the load on their cognitive system. They can also utilize writing to make cognitive adjustments (e.g., reflecting on the causes of exam anxiety, thinking about how to effectively cope with various difficult situations they may encounter during the exam, etc.) so that they can confront high-stress exam situations more comfortably [24,27].

Assisting test-anxious individuals with figuring out how to manage high-stress test settings and lessen the negative effects of stress on cognitive processing is of tremendous theoretical and practical importance. Despite the fact that several studies have indicated that EW can help subjects improve their cognitive processing performance in high-stress situations [23,27], previous studies have not addressed test-anxious individuals, nor have they compared the effects of EW interventions on individuals with different working memory capacities. Based on these analyses, the present study explores whether EW can help high test-anxious individuals reduce the degree of the CUP effect. In addition, the study examines whether the intervention effects are different in people with different working memory capacities.

## 2. Materials and Methods

### 2.1. Participants

We recruited subjects through a convenience sampling method among college students attending public classes at a university. We introduced our research project to students during the course and invited interested students to fill out a test anxiety inventory (TAI), which was used to screen subjects for the follow-up study. A total of 2436 students completed the TAI. A group of 469 students scoring in the top 20% of the scale were screened as high test-anxious subjects [28]. Of these, a total of 448 high test-anxious students completed the mathematical-operation–word memory-span task (i.e., OSPAN task) to measure working memory capacity [29,30], and their mean TAI score was 51.30 ± 5.99. Among them, 80 HWMs and 72 LWMs were selected according to their OSPAN task scores, which were one standard deviation above and below the mean, respectively. Finally, a total of 75 HWMs (28 males and 47 females, mean age 18.97 ± 0.81) and 63 LWMs (23 males and 40 females, mean age 19.11 ± 0.83) completed all of the experimental tasks. The OSPAN task scores of HWMs were 0.93 ± 0.06, and the scores of LWMs were 0.65 ± 0.08.

### 2.2. Measures

#### 2.2.1. Modular Arithmetic Task

The object of a modular arithmetic (MA) task is to judge the truth-value of problem statements such as “37 ≡ 21 (mod 4)”. Such a problem is solved by subtracting the middle number from the first number (i.e., 37 - 21) and then dividing this difference by the last number (i.e., 16/4). If the quotient is an integer (here 4), the statement is true. We set 2 difficulty levels for the MA task. Complex MA problems, such as 81 ≡ 17 (mod 6), are two-digit problems that require a borrow operation. Simple MA problems, such as 9 ≡ 5 (mod 2), are one-digit problems that do not require a borrow operation. Performance in MA tasks is closely related to working memory capacity [17]. Complex MA problems depend more on the working memory system than simple ones since they require the borrow operation and the two-digit operation. All subjects participating in the experiment were exposed to the MA tasks for the first time to avoid effects of practice on the experimental results.

#### 2.2.2. Test Anxiety Inventory

The test anxiety inventory comprises 20 items with scores ranging from 20 to 80. Participants rate the frequency of anxiety symptoms they experienced before, during, and after exams on a four-point scale. The scale has good validity and reliability [28]. The Cronbach alpha for this measure in the current study is 0.90.

#### 2.2.3. State Anxiety Inventory

The state–trait anxiety inventory (STAI) was developed by Spielberg [31]. We chose the state anxiety subscale to assess the participants’ immediate feelings of fear, tension, apprehension, and nervousness during the experiment. The state anxiety inventory comprises 20 items with scores ranging from 20 to 80. The Cronbach alpha for this measure in the current study is 0.88.

### 2.3. Experimental Flow

Participants needed to complete the MA task twice in the laboratory. The MA task was implemented on a PC using the software E-Prime (Psychology Software Tools Inc., Pittsburgh, PA, USA). Participants were instructed to judge MA problems as quickly as possible without sacrificing accuracy. Before the formal experiment, participants needed to finish six practice tests. In the formal experiment, there were 20 complex and 20 simple MA problems with half of the quotients being integers and half being non-integers. If the result was an integer, the participant pressed the “F”; if it was not an integer, the participant pressed the “J”. After completing the MA task, participants immediately filled out the state anxiety inventory.

One week later, the participants were invited back to the laboratory again to complete the high-stress MA task. According to the classical method that sets an evaluative stressful scenario [27,29], in this study we created a high-stress test situation by using the following instructions: “In the following experiment, you need to complete a standardized cognitive ability test. Your speed and accuracy in completing the test are the primary indicators used to calculate your score. Your score will be compared to those of other students, and the top 15% of participants will win a bonus of 100 CNY. You will be videotaped during the test to assess your performance objectively!” Next, the researcher turned on the camera.

Participants were given a randomly selected envelope with instructions for writing prior to beginning the high-stress MA task. The participants were divided into the EW group and the control group according to said instructions. Following that, participants needed to write for 10 min, complying with the instructions. To create a quiet and secure writing environment, the researcher left the lab to fetch something on purpose after the participant began writing. Ten minutes later, the researcher returned to the lab and gave the participant permission to complete the MA task. Participants filled out the state anxiety inventory directly after finishing the MA task.

### 2.4. Writing Instructions

The following instructions are for the EW group. Please take 10 min to write as thoroughly as possible about your feelings and thoughts about the upcoming test. In your writing, I want you to truly let yourself go and explore your feelings and thoughts as much as possible. Most importantly, you are encouraged to explore as much of your most profound emotional experience with the upcoming test as possible.

The following instructions are for the control group. Please take 10 min to objectively describe what you experienced during the past 24 h. For instance, the classes you attended, the sports you played, the people you met, etc. Please describe as objectively as possible, without mentioning your emotions, feelings, or opinions.

### 2.5. Written Content Analysis

The Chinese psychoanalysis system (CPAS) was employed in this study to analyze the content of writing [32]. The CPAS was developed by the Institute of Psychology at University of Chinese Academy of Sciences. It has the ability to count the proportion of dozens of word types and analyze the features of various word types in Chinese text. In this study, we counted the percentage of emotion words, positive emotion words, negative emotion words, anxiety words, cognitive words, causal words, and insight words, as well as the total number of words.

### 2.6. Statistical Analyses

All statistical tests were conducted using SPSS 24 (IBM, Armonk, NY, USA). All tests were two-tailed, and significance levels were evaluated using an alpha of 0.05. Descriptive statistics present mean values of experimental data with standard deviation (SD). Independent samples *t*-tests were used to analyze the differences in written content between the EW and the control group. A repeated measures ANOVA was performed to compare the CUP effect under the various writing conditions. Group (EW group, control group) and working memory capacity (high, low) were used as between-subject factors, while pressure level (low-stress, high-stress) was utilized as a within-subject factor. The relationship between the written content and the CUP effect was analyzed using partial correlation analysis.

## 3. Results

The statistical results of the written content of the EW group and the control group are shown in Table 1. Independent samples *t*-test showed that the EW group wrote significantly higher proportions of cognitive words, insight words, causal words, emotion words, negative emotion words, and anxiety words than the control group. However, there was no statistically significant difference between the EW group and the control group in the percentage of positive emotion words. These results suggest that the EW group achieved emotional disclosure through writing (especially more negative and anxious emotions) and made more cognitive adjustments than the control group.

Subjects’ state anxiety scores during the experiment are shown in Table 2. With state anxiety scores as the dependent variable, a repeated-measures ANOVA of 2 (group: EW group, control group) × 2 (working memory capacity: high, low) × 2 (pressure level: low-stress, high-stress) was conducted. The results showed that the main effect of pressure level was significant (F(1, 134) = 50.44, *p* = 0.000, *η_p_*^2^ = 0.27), which indicated the participants had significantly higher state anxiety in high-stress situations. The group × pressure level interaction was significant (F(1, 134) = 14.09, *p* = 0.000, *η_p_*^2^ = 0.10). Simple effects analysis showed no significant difference in state anxiety between the two groups in the low-stress condition (*t*(136) = 0.56, *p* = 0.58, *Cohen’d* = 0.10), but the control group had a significantly higher level of state anxiety than the EW group in the high-stress condition (*t*(136) = −3.61, *p* = 0.000, *Cohen’d* = 0.62). In addition, the main effects of group (F(1, 134) = 3.16, *p* = 0.08, *η_p_*^2^ = 0.23), and working memory capacity (F(1, 134) = 0.44, *p* = 0.51, *η_p_*^2^ = 0.003), the group × working memory capacity interaction (F(1, 134) = 0.19, *p* = 0.67, *η_p_*^2^ = 0.001), the working memory capacity × pressure level interaction (F(1, 134) = 1.20, *p* = 0.28, *η_p_*^2^ = 0.009), and the three-factor interaction (F(1, 134) = 0.02, *p* = 0.90, *η_p_*^2^ = 0.000) were not significant. The results described above suggest that the EW intervention was helpful for high test-anxious subjects to alleviate their state anxiety levels when facing high-stress situations.

Subjects’ scores in the MA task are shown in Table 2. With the accuracy of complex MA task as the dependent variable, a 2 (group: EW group, control group) × 2 (working memory capacity: high, low) × 2 (pressure level: low-stress, high-stress) repeated-measures ANOVA was conducted. The results showed that the main effect of the group was not significant (F(1, 134) = 1.16, *p* = 0.28, *η_p_*^2^ = 0.009). The main effect of working memory capacity was significant (F(1, 134) = 40.35, *p* = 0.000, *η_p_*^2^ = 0.23), indicating the accuracy was significantly higher in HWMs. The main effect of pressure level was significant (F(1, 134) = 14.98, *p* = 0.000, *η_p_*^2^ = 0.10), with significantly lower accuracy in the high-stress test than in the low-stress test. The group × working memory capacity interaction was not significant (F(1, 134) = 2.43, *p* = 0.12, *η_p_*^2^ = 0.02), whereas the group × pressure level interaction (F(1, 134) = 6.28, *p* = 0.01, *η_p_*^2^ = 0.05), the working memory capacity × pressure level interaction (F(1, 134) = 15.05, *p* = 0.000, *η_p_*^2^ = 0.10), and the three-factor interaction (F(1, 134) = 4.00, *p* = 0.048, *η_p_*^2^ = 0.03) were significant. A simple-effect analysis of the three-factor interaction was conducted. For LWMs, the main effect of the group, the pressure level, and the group × pressure level interaction were all not significant (Fs < 1, *p*s > 0.05), showing that the MA task performance of LWMs was not affected by the stressful scenario. For HWMs, the main effect of group (F(1, 73) = 85.07, *p* = 0.000, *η_p_*^2^ = 0.54), and the group × pressure level interaction were significant (F(1, 73) = 28.74, *p* = 0.000, *η_p_*^2^ = 0.28). Further analysis showed that there was no significant accuracy difference between high-stress and low-stress tests in the EW group (*t*(35) = 1.13, *p* = 0.28, Cohen’s *d* = 0.73), whereas accuracy in the high-stress test was significantly lower than in the low-stress test in the control group (*t*(38) = 8.87, *p* = 0.000, Cohen’s *d* = 1.22). The results described above indicate that HWMs showed the CUP effect in the high-stress test, but EW could reduce the negative effect of test stress.

With the accuracy of the simple MA task as the dependent variable, a 2 (group: EW group, control group) × 2 (working memory capacity: high, low) × 2 (pressure level: low-stress, high-stress) repeated-measures ANOVA was conducted. The results showed that the main effect of working memory capacity was significant (F(1, 134) = 11.33, *p* = 0.001, *η_p_*^2^ = 0.08), indicating that the accuracy of HWMs was significantly higher than those of the LWMs. The main effect of group (F(1, 134) = 2.50, *p* = 0.12, *η_p_*^2^ = 0.02), the main effect of pressure level (F(1, 134) = 0.02, *p* = 0.90, *η_p_*^2^ = 0.000), the group × working memory capacity interaction (F(1, 134) = 1.35, *p* = 0.25, *η_p_*^2^ = 0.01), the group × pressure level interaction (F(1, 134) = 0.42, *p* = 0.52, *η_p_*^2^ = 0.003), the working memory capacity × pressure level interaction (F(1, 134) = 2.12, *p* = 0.15, *η_p_*^2^ = 0.02), and the three-factor interaction (F(1, 134) = 0.26, *p* = 0.61, *η_p_*^2^ = 0.002) were not significant. These results suggest that neither the pressure level nor the EW intervention affected the simple MA task performance.

Using the total number of words written by each participant as a control variable, a partial correlation analysis was conducted between the CUP value (the difference between the accuracy of the MA task in low- and high-stress situations) and the proportion of words that were written (Table 3). The results showed that the CUP value of the complex task was significantly and negatively correlated with the proportion of emotional words and cognitive words. That is, the higher the proportion of emotional words and cognitive words used in writing, the lower the degree of the CUP effect in the complex MA task. In addition, there was no significant correlation between the written content and the CUP value in the simple MA task.

## 4. Discussion

This study examined the performance of high test-anxious individuals in MA tasks under high-stress and low-stress conditions in order to investigate the impact of EW interventions on the CUP effect in HWMs and LWMs. The results showed that EW interventions were able to alleviate the state anxiety levels of high test-anxious subjects when exposed to high-stress situations. HWMs demonstrated the CUP effect when completing complex MA tests under high-stress conditions, and EW could help them reduce the severity of the CUP effect. In contrast, the performance of LWMs was not affected by the high-stress condition and they did not show the CUP effect. Furthermore, HWMs and LWMs were both not affected by high-stress conditions in simple MA tests.

The present study indicated that EW interventions helped high test-anxious individuals with high working memory capacity to alleviate state anxiety and CUP effect. Previous research has shown that participants who use more cognitive words during writing significantly increase their working memory capacity [25,26] and minimize intrusive and avoidant thoughts about bad occurrences [21,22,33]. The present study supports these ideas and shows that the high test-anxious participants who used more emotional and cognitive words in the written content were less affected by test stress. Emotion words are a marker of emotional catharsis in EW interventions [20]. High test-anxious participants can release negative test-related emotions to lessen the stressful experience during the exam and alleviate the burden on the cognitive system [27]. The cognitive words can reflect the writer’s cognitive adjustment process [21,22]. High test-anxious participants can adjust their irrational cognition to the test, analyze the reasons for their test anxiety, and think about how to confront the test better through EW interventions [23]. Therefore, EW interventions can help high test-anxious individuals to release emotions and adjust cognition, thereby serving to reduce task-irrelevant thinking and attenuating the CUP effect [19]. The EW intervention is simple and convenient to operate, with no special requirements for the location or timing, making it suitable for dissemination on campus. High test-anxious students can reduce anxiety and even improve academic performance simply by following instructions to fully express their feelings [34].

Notably, EW interventions did not significantly affect the performance of LWMs in completing complex MA task in the present study. Consistent with previous findings, this study showed that LWMs’ performance was not affected by the high-stress test scenario and they did not exhibit the CUP effect [15]. When completing complex MA tasks, LWMs tend to employ an association-based processing strategy and rely less on the working memory system [17,18]. Although EW interventions decreased LWMs’ state anxiety, they had no significant impact on their MA task performance. It is evident that there are individual differences in the effects of EW interventions on cognitive function. EW interventions can help test-anxious subjects (both HWMs and LWMs) reduce state anxiety when facing stressful test situations, indicating that EW is very effective in relieving test anxiety [27,35]. In practical application, educators or counselors can recommend EW to high test-anxious students to help them relieve their anxiety. Additionally, educators can use working memory assessments to assess the test-anxious students’ working memory capacity and encourage those HWMs to take part in EW interventions in order to alleviate CUP effects.

The results of this study should be considered in light of several limitations. First, we did not measure the CUP effect in a realistic evaluative environment. We created high-stress test situations in the laboratory through cash rewards, peer comparisons, and video recordings, but there were differences between the psychological and cognitive effects of the simulated high-stress situations and real-life major examinations on high test-anxious individuals. Second, this study did not track the long-term effects of writing expression. Accordingly, future research is needed to determine whether EW has the potential to substantially help high test-anxious students in a real-life evaluative situation.

## 5. Conclusions

The current study demonstrated that EW interventions were effective in reducing state anxiety levels and attenuating the detrimental effects of test stress on cognitive processing in test-anxious individuals with high working memory capacity. Educators might consider EW as a convenient method to address test anxiety in various types of schools.

## Figures and Tables

**Table 1:** Analysis of the written content of the EW group and the control group (M±SD).

	EW Group (*n* = 67)	Control Group (*n* = 71)	*t*	*p*
Emotion words	11.80 ± 3.92	6.10 ± 2.92	9.70	0.000
Positive emotion words	4.94 ± 2.84	4.44 ± 2.52	1.10	0.28
Negative emotion words	4.29 ± 2.69	2.82 ± 2.04	3.62	0.000
Anxiety words	2.63 ± 1.56	1.28 ± 1.03	6.04	0.000
Cognitive words	32.31 ± 9.11	19.85 ± 5.80	9.63	0.000
Insight words	7.67 ± 3.10	3.93 ± 3.03	7.16	0.000
Causal words	2.23 ± 1.11	1.53 ± 1.09	3.74	0.001
Total number of words	150.95 ± 70.04	156.09 ± 63.84	−0.45	0.65

Note: In addition to the total number of words, the descriptive statistics data in the table indicate the percentage of various word types compared to the total number of words in the written content.

**Table 2:** MA task accuracy and state anxiety of subjects in the EW and the control group (M±SD).

Group.	Working Memory Capacity	Pressure Level	Complex MA	Simple MA	State Anxiety
EW group (*n* = 67)	High (*n* = 36)	Low-stress	0.98 ± 0.03	0.98 ± 0.03	41.17 ± 7.88
		High-stress	0.95 ± 0.05	0.98 ± 0.03	42.97 ± 7.63
		CUP value	0.03 ± 0.05	0.003 ± 0.04	
	Low (*n* = 31)	Low-stress	0.88 ± 0.15	0.95 ± 0.10	40.16 ± 7.44
		High-stress	0.88 ± 0.06	0.96 ± 0.07	43.45 ± 7.89
		CUP value	0.00 ± 0.14	−0.01 ± 0.04	
Control group (*n* = 71)	High (*n* = 39)	Low-stress	0.99 ± 0.02	0.99 ± 0.02	40.92 ± 7.94
		High-stress	0.88 ± 0.09	0.98 ± 0.03	48.26 ± 8.88
		CUP value	0.12 ± 0.08	0.02 ± 0.04	
	Low (*n* = 32)	Low-stress	0.89 ± 0.12	0.97 ± 0.06	38.75 ± 8.70
		High-stress	0.88 ± 0.11	0.98 ± 0.04	47.94 ± 7.55
		CUP value	0.00 ± 0.14	−0.01 ± 0.07	

Note: CUP value = MA accuracy in high-stress test subtracted from MA accuracy in low-stress test. Higher values indicate stronger CUP effect.

**Table 3:** Partial correlation analysis of written content and the CUP values of the MA task (*r*).

	Emotion Words	Positive Emotion Words	Negative Emotion Words	Anxiety Words	Cognitive Words	Insight Words	Causal Words
Complex MA	−0.18 *	−0.17	0.01	−0.08	−0.24 **	−0.11	−0.07
Simple MA	−0.04	−0.14	−0.02	−0.05	−0.16	−0.02	−0.03

* *p* < 0.05, ** *p* < 0.01.

## Data Availability

The datasets collected and analyzed during the current study are available from the corresponding author on reasonable request.
